# Impulsivity Derived From the Dark Side: Neurocircuits That Contribute to Negative Urgency

**DOI:** 10.3389/fnbeh.2019.00136

**Published:** 2019-06-25

**Authors:** Eric P. Zorrilla, George F. Koob

**Affiliations:** ^1^Department of Neuroscience, The Scripps Research Institute, La Jolla, CA, United States; ^2^Neurobiology of Addiction Section, Intramural Research Program, National Institute on Drug Abuse, Baltimore, MD, United States

**Keywords:** negative urgency, impulsivity, compulsive drug use, negative affect, withdrawal, substance or alcohol use disorder, orbitofrontal cortex, extended amygdale

## Abstract

Negative urgency is a unique dimension of impulsivity that involves acting rashly when in extreme distress and impairments in inhibitory control. It has been hypothesized to derive from stress that is related to negative emotional states that are experienced during the *withdrawal/negative affect* stage of the addiction cycle. Classically, a transition to compulsive drug use prevents or relieves negative emotional states that result from abstinence or stressful environmental circumstances. Recent work suggests that this shift to the “dark side” is also implicated in impulsive use that derives from negative urgency. Stress and anxious, depressed, and irritable mood have high comorbidity with addiction. They may trigger bouts of drug seeking in humans *via* both negative reinforcement and negative urgency. The neurocircuitry that has been identified in the “dark side” of addiction involves key neuropeptides in the central extended amygdala, including corticotropin-releasing factor. The present review article summarizes empirical and conceptual advances in the field to understand the role of the “dark side” in driving the risky and detrimental substance use that is associated with negative urgency in addiction.

## Impulsivity, Compulsivity, and Negative Urgency

**Figure 1:**
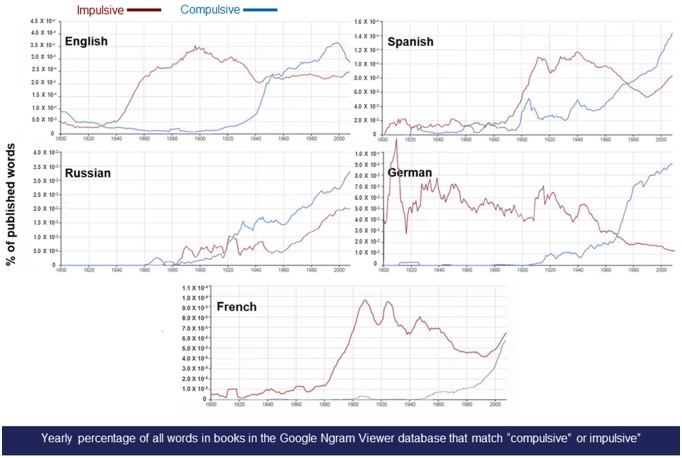
Changes in use of the terms compulsive and impulsive in literature over time. Trends in the use of the terms “impulsive” and “compulsive” across languages. Panels show standardized *n*-gram frequency relative to the corpus of published 1-g in Google Books for that language (Michel et al., 2011). Across languages, *n-grams* that translate into “compulsive” sharply grew in relative use during the 20th century. The *n-gram* datasets were generated in July 2012 by Google. Searches were performed in October 2018 with http://books.google.com/ngrams.

Impulsivity and compulsivity play central roles in both lay and scientific conceptualizations of addiction (Jentsch and Taylor, 1999; Koob and Le Moal, 2008; Economidou et al., 2009; Kwako et al., 2019). Behavior that is impulsive, from the Latin *impuls*- (“tending to impel or driven onward”), is defined as an action that is instigated suddenly without forethought of potential consequences. An impulsive behavior may be experienced as acting instinctively upon emotions or involuntary impulses and can be followed by regret, guilt, or shame. Externally, impulsivity may appear as acting hastily, capriciously, or on whims and prioritizing immediate gains vs. later outcomes. Behavior that is compulsive, from the Latin *compuls*- (“driven or forced”), is defined as an action that results from or is related to an irresistible urge, whereby irresistibility can be operationalized as behavior that persists despite aversive or incorrect outcomes. A compulsive behavior is often experienced as outside of one’s control and, even during its performance, can be intrusive, unwanted, and ego-dystonic. From a historical perspective, the constructs of impulsivity and compulsivity have received increasing cross-cultural attention recently, much focused on their role in addictive behavior. The word “impulsive” exploded into use in Western countries generally during the later 19th century and preceded the emergence of the word “compulsivity” during the later 20th century (Michel et al., 2011; Figure 1). Many compulsive behaviors are commonly described (Table 1), but “compulsive drug use” and “compulsive drinking” are among the most frequent, constituting ~1.3% of the written use of “compulsive” in the modern English language (Table 1). Here, we propose that the even more modern construct of negative urgency (Cyders and Smith, 2008) may play an integrative role that bridges impulsivity with compulsivity in the “dark side” of addiction.

**Table 1:** Relative *n*-gram frequency (vs. “compulsive”) in the English Corpus of Google Books.

Term	Peak Use (1940–2008)	Year 2000
Compulsive eating	2.1% (1988)	1.5%
Compulsive gambling	1.8% (1992)	1.6%
Compulsive shopping^1^	1.4% (2008)	0.93%
Compulsive drug use^2^	0.70% (1984)	0.50%
Compulsive drinking^3^	0.64% (1958)	0.20%
Compulsive washing/cleaning^4^	0.26% (2008)	0.22%
Compulsive hoarding	0.25% (2008)	0.15%
Compulsive exercise	0.23% (2007)	0.20%
Compulsive checking	0.23% (2008)	0.17%
Compulsive sex	0.16% (1996)	0.16%
Compulsive grooming^5^	0.11% (2004)	0.10%
Compulsive Internet use^6^	0.05% (2008)	0.03%

*Note: searches were performed in May 2018 with http://books.google.com/ngrams as [(term)/compulsive]. The n-gram dataset was generated in July 2012 by Google (v20120701). ^1^Includes compulsive shopping, compulsive buying. ^2^Includes compulsive drug, compulsive substance, compulsive smoking, compulsive nicotine, compulsive tobacco, compulsive marijuana, compulsive caffeine, compulsive cocaine, compulsive opiate, compulsive heroin, compulsive morphine, compulsive psychostimulant, compulsive amphetamine, compulsive methamphetamine. ^3^Includes compulsive drinking, compulsive alcohol, compulsive ethanol. ^4^Includes compulsive washing, compulsive hand washing, compulsive cleaning. ^5^Includes compulsive grooming, compulsive hair, compulsive nail. ^6^Includes compulsive Internet, compulsive video game*.

## Definition and Measurement of Negative Urgency

Conceived as an emotion-based trait, negative urgency refers to acting rashly when in extreme distress and involves impairments in inhibitory control (Cyders and Smith, 2008). In people, negative urgency has been measured *via* the eponymous subscale of the Urgency, Premeditation (lack of), Perseverance (lack of), Sensation Seeking, Positive Urgency, Impulsive Behavior Scale (UPSS-P; Whiteside and Lynam, 2003; Lynam et al., 2006; Cyders and Smith, 2008). The 12 self-report Negative Urgency items, distinct from the more recently developed Positive Urgency subscale, quantify the tendency to act rashly during negative affective states. Exemplar items that refer to actions during negative mood include the following: (i) When I feel bad, I will often do things I later regret in order to make myself feel better now; (ii) I often make matters worse because I act without thinking when I am upset; and (iii) Sometimes when I feel bad, I can’t seem to stop what I am doing even though it is making me feel worse. Although the first and second of these items are consistent with the reviewed definition of impulsive behavior, the third is also consistent with compulsivity (i.e., persistent “irresistibly” despite negative outcomes).

Other drug use-relevant items that have been identified by factor analysis to also load on Negative Urgency include the following: (i) I have trouble controlling my impulses; (ii) I have trouble resisting my cravings (for food, cigarettes, etc.); and (iii) It is hard for me to resist acting on my feelings. Each of these items is potentially consistent with either impulsivity or compulsivity, depending on their perseveration in the face of actual negative or incorrect outcomes. Thus, although Negative Urgency is typically viewed as a dimension of impulsive behavior, several of the items that measure it may also detect a predisposition for or changes in compulsive behavior (i.e., irresistible, viscerally driven behavior with loss of control despite negative consequences).

In early work, the Negative Urgency subscale showed good internal consistency and construct validity (Whiteside and Lynam, 2003). Subsequently, the UPSS-P has been adopted for a short form (Cyders et al., 2014b) and for children (Zapolski et al., 2011) and has been translated to many languages (Van der Linden et al., 2006; Kämpfe and Mitte, 2009; Keye et al., 2009; Verdejo-García et al., 2010; Billieux et al., 2012; Candido et al., 2012; Lim and Lee, 2014; D’Orta et al., 2015; Fossati et al., 2016; Poprawa, 2016; Shokri and Sanaeeour, 2016; Bteich et al., 2017; Sediyama et al., 2017; Bousardt et al., 2018).

## Neurocircuitry of Addiction: View From the Dark Side

Preclinical research in animal models and imaging studies in humans have provided critical insights into the pathological behavior that characterizes addiction. Convergent results show that individuals with addiction undergo progressive functional and even structural disruptions of brain regions that subserve normal processes of incentive salience, habits, emotional regulation, stress, and executive function (Robbins and Everitt, 1999; Everitt and Robbins, 2005; Koob and Volkow, 2010, 2016; Goldstein and Volkow, 2011; Belin et al., 2013). Heuristically, drug addiction has been conceptualized as a cycle of three stages. Each stage reflects basic neurocircuitry that is involved in aberrant motivation, and each stage is predominantly linked to a functional domain and brain functional networks that interact with each other (Figure 1). The *binge/intoxication* stage, *via* the neurocircuitry of the basal ganglia, reflects the rewarding, incentive salience, and pathological habit effects of drugs. The *withdrawal/negative affect* stage, *via* the extended amygdala and other regions (e.g., lateral habenula), reflects the loss of reward and motivation and the enhanced sensitivity and recruitment of brain stress systems, leading to negative emotional symptoms, such as dysphoria, anhedonia, and irritability (Figure 1). The *preoccupation/anticipation* (“craving”) stage, *via* neurocircuitry of the prefrontal cortex (PFC), reflects deficits in executive function, including impulsivity and the loss of control over drug taking.

The neurocircuitry and neuropharmacology of the *withdrawal/negative affect* stage of the addiction cycle is built on the opponent-process, affective dysregulation model of addiction (Koob and Bloom, 1988; Koob and Le Moal, 2005, 2008; Koob and Zorrilla, 2010; Zorrilla et al., 2013, 2014; George et al., 2014; Koob et al., 2014), an extension of opponent-process theory (Solomon and Corbit, 1974; see “Glossary” section for definitions of relevant terms in the model). The “dark side” affective dysregulation hypothesis posits that drugs of abuse initially activate brain circuits that elicit pleasurable emotional states (“a-process”). To restore emotional homeostasis, however, counterregulatory, opponent processes (“b-process”) follow that decrease mood and increase negative emotional states, such as vigilance and tension.

The affective dysregulation model extends opponent-process theory by further proposing that hysteresis develops with repeated cycles of intoxication/withdrawal whereby the negative opponent process (b-process) initiates earlier, to a greater degree and more persistently than the rewarding a-process. With repeated exposure to the substance of abuse, the opponent process eventually predominates. A greater quantity and frequency of use of the substance is then needed to restore euthymia. When the substance is not used, negative emotional symptoms of withdrawal develop, such as irritability, anxiety, dysphoria, and subjective feelings of need. This deficit emotional state or hyperkatifeia (Shurman et al., 2010) is putatively dissociable from somatic withdrawal signs and can sensitize and persist with repeated substance use. This model accounts for protracted abstinence symptoms, in which hypohedonia, negative emotional behavior, hyperarousability, and greater behavioral responses to stress may be seen despite months of sustained abstinence. Under this conceptualization of the “dark side” of addiction (Koob and Le Moal, 2001, 2005, 2008), substance use is compulsively escalated or renewed (in relapse) *via* negative reinforcement mechanisms because it transiently prevents or relieves the emotional sequalae of the *withdrawal/negative affect* stage. Thus, this allostatic model of brain motivational systems views addiction as a cycle of increasing dysregulation of brain reward/anti-reward mechanisms that yield negative emotional states and thereby promote compulsive drug use *via* a different source of motivation, namely negative reinforcement (Koob and Bloom, 1988).

The counteradaptive b-processes that initially function to restore emotional homeostasis but ultimately lead to allostasis are proposed to reflect two mechanisms: within-system downregulation of brain reward/incentive motivational systems and between-system recruitment/activation of stress/aversion neurocircuitry (Koob and Le Moal, 2008). With regard to within-system neuroadaptations, one prominent hypothesis is that dopamine and opioid peptide reward/incentive motivational systems become compromised, which is evident during both acute withdrawal and protracted abstinence. The argument is that a decrease in dopamine and opioid peptide function leads to lower motivation for non-drug-related stimuli and greater sensitivity to cues that are associated with the abused drug (i.e., increase in incentive salience; Melis et al., 2005). Supporting this hypothesis, animal models of alcohol withdrawal show a decrease in activity of the mesolimbic dopamine system and a decrease in serotonergic neurotransmission in the nucleus accumbens (Koob, 2015). Strong support for a compromised mesolimbic dopamine system is seen in both animal studies and human imaging studies (Ashok et al., 2017). Other proposed within-system neuroadaptations may include changes in gene transcription and receptor transduction mechanisms in the connections of the mesolimbic dopamine system, including the nucleus accumbens (Feng and Nestler, 2013).

With regard to between-system neuroadaptations that underlie the *withdrawal/negative affect* stage, neurocircuits and neurochemical systems that subserve arousal-stress putatively become engaged in a homeostatic attempt to counter the ongoing presence of the perturbing drug and restore normal emotional function. The extended amygdala (Heimer and Alheid, 1991) may represent a common neuroanatomical substrate that integrates brain arousal-stress and hedonic processing systems to produce the proposed between-system adaptations. The extended amygdala, a distinct entity in the basal forebrain (Alheid and Heimer, 1988), is composed of several basal forebrain structures, including the central nucleus of the amygdala, bed nucleus of the stria terminalis, sublenticular substantia innominata, and a transition zone in the medial nucleus accumbens (e.g., shell; Heimer and Alheid, 1991). The extended amygdala is innervated by many limbic structures, including the basolateral amygdala and hippocampus, and sends major projections to the medial ventral pallidum and lateral hypothalamus. It thus bridges classic limbic structures with the extrapyramidal motor system. Accordingly, the extended amygdala is thought to play important roles in both fear conditioning (Le Doux, 2000) and emotional aspects of pain processing (Neugebauer et al., 2004).

Consistent with the posited between-system engagement of brain arousal-stress systems, chronic exposure to all major drugs with dependence or abuse potential leads to the dysregulation of corticotropin-releasing factor (CRF) brain stress systems, including those in the hypothalamic-pituitary-adrenal axis and extended amygdala. Thus, acute withdrawal from chronic drug exposure yields higher levels of adrenocorticotropic hormone, corticosterone, and extracellular amygdala CRF (Koob, 2008). In addition to CRF, dynorphin, vasopressin, norepinephrine, hypocretin (orexin), and substance P may also contribute to negative emotional states during drug withdrawal. Thus, multiple neuropeptides and neurotransmitters underlie the activation of pro-stress, pro-negative emotional state neurocircuitry and form the neurochemical bases for hedonic opponent processes (Koob, 2015). In opposition, anti-stress circuitry, including neuropeptide Y (NPY), nociceptin, and endocannabinoid systems (Koob, 2015), normally buffer the aforementioned pro-stress actions. Thus, the activation of pro-stress neuromodulators coupled with an inadequate anti-stress response may yield negative emotional states that drive negative reinforcement.

## Negative Urgency and Addiction: Tobacco, Alcohol, Cocaine, Pathological Gambling, and Food

Negative affect, negative urgency (VanderVeen et al., 2016), and attentional (“decreased ability to focus”) and motor (“acting without thinking”) impulsivity are comorbid in alcohol and drug addiction (Billieux et al., 2007; Stautz and Cooper, 2013). Indeed, negative urgency may be an endophenotype for alcohol and tobacco addiction and other clinical disorders (Cyders and Smith, 2008; VanderVeen et al., 2016). Negative urgency has been particularly associated with negative emotional states (VanderVeen et al., 2016). Negative urgency is viewed as contributing to addictive behavior under this affective dysregulation, negative reinforcement framework because: (1) negative emotional episodes (i.e., irritability, anxiety, and depression) that are associated with the *withdrawal/negative affect* stage may represent or give rise to intense urges (e.g., to attempt to self-medicate) that are moderated or mediated by negative urgency; and (2) the impairment of executive control that is associated with negative urgency may decrease the capacity to resist urges to pursue substance use in the *preoccupation/anticipation* stage such that behavior occurs both rapidly without forethought of potential harm (impulsively) and also, with disease progression, despite actual negative or incorrect consequences (compulsively).

### Tobacco

Consistent with a negative reinforcement model, smoking to relieve negative mood is a common self-reported motivation for smokers (Doran et al., 2009). Expectancies of smoking’s effects on emotion are linked to the broad construct of impulsivity (Doran et al., 2007a,b, 2011). Previous work has hypothesized a unique role for the negative urgency aspect of impulsivity specifically in predicting levels of nicotine dependence. Indeed, a meta-analysis that explored the relationships between impulsivity-related traits and both smoking status and nicotine dependence severity found that negative urgency’s relationship to the severity of nicotine dependence was the second strongest one identified (Kale et al., 2018). One model, termed the Acquired Preparedness model, of addictive behaviors, posits that individuals are differentially prepared to acquire high-risk expectancies of smoking’s effects based on individual differences in personality. These include predispositions to act impulsively when in extreme negative mood states (Negative Urgency) or positive mood states (Positive Urgency; Smith and Anderson, 2001). The model proposes that individuals who act out in response to extreme emotional states are more likely to perceive smoking (or similar behaviors) as reinforcing compared with individuals who do not act out in response to extreme emotional states. They then form expectancies for the positive and negative reinforcement that would result from smoking. For positive reinforcement with positive urgency, individuals may learn that smoking is enjoyable and pleasurable, an expectancy that would lead to smoking when in a good mood and positive reinforcement from smoking. For negative reinforcement with negative urgency, individuals may instead learn that smoking will lessen their negative emotional state. This expectancy may lead them to smoke to alleviate such states and promote negative reinforcement processes.

Consistent with the Acquired Preparedness model, positive affect expectancies for smoking in 139 adult college-aged smokers were found to mediate the relationship between positive urgency and the degree of nicotine dependence symptoms (Spillane et al., 2013). Conversely, the study also found an indirect association between negative urgency and the expectancy that smoking would reduce negative affect whereby affect relief expectancies for smoking mediated the relationship between negative affect and levels of nicotine dependence (Spillane et al., 2013). More specifically, negative urgency explained the variance of negative affect reduction expectancies, which in turn accounted for the variance in smoking (Spillane et al., 2013). In an earlier study (Spillane et al., 2010), negative urgency did not predict the level of dependence. However, both studies included subjects with low-to-moderate nicotine dependence, and the authors hypothesized that negative urgency may manifest in other domains of smoking behavior. For example, high-negative-urgency individuals are predicted to be at greater risk for relapsing in response to the negative emotional states that are associated with quitting.

Consistent with this observation, a study of smokers with high anxiety sensitivity found that these subjects tended to show negative urgency, which in turn was associated with greater expectations that negative reinforcement (i.e., affect relief) would result from smoking or abstaining from smoking (Guillot et al., 2014). Others showed that the expectancy that smoking would reduce negative affect mediated the relationship between negative urgency and the level of nicotine dependence in college-aged smokers (Spillane et al., 2013). The authors argued that treatments that target the fear of anxiety symptoms and the tendency to act impulsively in response to negative affect may be particularly efficacious in promoting smoking cessation in smokers with high anxiety sensitivity (Guillot et al., 2014). Examples include interoceptive exposure, distress tolerance skills training, and mindfulness training.

### Alcohol

Data also support a role for negative urgency in alcohol use disorders (AUDs). One study examined within-person relationships among specific emotions, alcohol intoxication, and acute dependence symptoms and between-person effects of urgency vs. self-control (i.e., premeditation and perseverance). Consistent with the above-reviewed negative reinforcement model (Koob and Bloom, 1988; Baker et al., 2004; Ahmed and Koob, 2005), the authors found that sadness and anxiety were each directly associated with dependence symptoms and that, in some participants, daytime anxiety was positively associated with subsequent alcohol intoxication (Simons et al., 2010). Relevant to the present discourse, the relationship between anxiety and intoxication was significant only for individuals who were higher in negative urgency or lower in positive urgency (Simons et al., 2010).

A study of 215 undergraduate students who indicated a history of deliberate self-harm tested the hypothesis that negative urgency accounts for the effects of affective lability and self-control on self-harm, problematic alcohol consumption, and eating problems. Accordingly, structural equation modeling found that negative urgency was significantly associated with several self-harming measures, problematic alcohol use, and eating problems (Dir et al., 2013). Negative urgency was also directly associated with affective lability and inversely associated with self-control (Dir et al., 2013). The authors asserted that negative urgency was unique in being the only impulsivity-related trait in their study that increased the risk of self-harm and problematic eating or alcohol use (Dir et al., 2013).

Another study explored the way in which negative urgency was related to emotional experience and alcohol-seeking behaviors in 34 community-dwelling, alcohol-using adults. The volunteers were tested in two counterbalanced sessions of intravenous alcohol self-administration: one with negative mood induction and one without (VanderVeen et al., 2016). Negative urgency predicted greater mood changes in response to negative mood induction and greater alcohol craving before and after an alcohol prime selectively in the negative mood condition (and not in the neutral mood condition). The subjects also had higher blood alcohol levels and more alcohol seeking. Thus, the results suggest that negative urgency could amplify alcohol self-administration *via* an increase in emotional reactivity to negative events and an increase in alcohol craving in response to an initial alcohol exposure (VanderVeen et al., 2016).

A study of 194 college students investigated whether the relationship between negative urgency and drinking behavior could be explained by positive and negative alcohol outcome expectancies and drinking motive. Path analysis showed indirect relationships between negative urgency and alcohol use *via* alcohol outcome expectancies and affect enhancement motives (Anthenien et al., 2017). The authors hypothesized that individuals who present high negative urgency may drink alcohol to reverse their emotional distress by enhancing their positive affect (enhancing positive alcohol outcome expectancies; Anthenien et al., 2017).

Negative urgency has also been linked to the higher prevalence of comorbid AUD in adults with attention-deficit/hyperactivity disorder (ADHD; Daurio et al., 2018). In a study of 794 adult subjects, different components of impulsivity were tested to understand the relationship between ADHD symptoms and the severity of alcohol dependence. Negative and positive urgency mediated the relationship between alcohol dependence severity and overall adult ADHD symptoms, including hyperactivity/restlessness and problems with self-concept (Daurio et al., 2018). The authors suggested that negative and positive urgency account for more of the relationship between alcohol dependence severity and ADHD symptoms than do other common measures of impulsivity, such as sensation seeking, the lack of premeditation, and the lack of perseverance.

A study of 273 young-adult Australians examined the contributions of several different facets of impulsivity to problematic alcohol use. Negative urgency, like many of the other measures of impulsivity, was directly related to greater alcohol intake, binge drinking, and alcohol-related problems. When simultaneously considered as regression predictors, however, only negative urgency and the lack of premeditation emerged as unique predictors of binge drinking behavior. Similarly, only negative urgency and positive urgency were unique predictors of alcohol-related problems. Both effects of negative and positive urgency were similarly present in college-attending vs. non-college-attending participants. The authors concluded that interventions that are tailored to target impulsive responding in response to extreme negative or positive emotional states are needed to mitigate binge drinking and adverse drinking outcomes in young adults (Tran et al., 2018).

A study of 675 community-dwelling adults in the Rockland Project used structural equation modeling path analysis to evaluate the mediating vs. moderating roles of urgency in the relationship between depression and problematic alcohol or cannabis use. Negative urgency, not positive urgency, was a unique mediator of the relationships between depressive symptoms and both problematic alcohol use and problematic cannabis use. Additionally, negative urgency moderated the relationship between depressive symptoms and problematic cannabis use. Specifically, at low levels of negative urgency, depressive symptoms predicted less problematic cannabis use, whereas at high levels of negative urgency, depressive symptoms predicted greater cannabis use. The authors concluded that despite being statistically correlated with each another, negative and positive urgency had distinct influences on the relationship between depressive symptoms and alcohol and cannabis use, with negative urgency having unique predictive significance (Um et al., 2019a).

A recent study sought to identify core functional domains that are associated with AUD in a large, diverse sample of 454 volunteers. Factor analysis identified three intercorrelated functional dimensions—impaired executive control, negative emotionality, and dysphoria-associated incentive salience—each of which discriminated participants with vs. without AUD. Negative urgency was 50% higher in participants with AUD than those without AUD and loaded strongly on the impaired inhibitory control factor and, less so, on the negative emotionality factor (Kwako et al., 2019).

### Cocaine

For studies of cocaine addiction, evidence indicates an interaction between negative urgency and cognitive performance. In a study that compared cocaine-dependent individuals and pathological gamblers with regard to cognitive performance and impulsivity, cocaine-dependent individuals had higher scores on UPPS-P Negative Urgency and poorer working memory performance (Albein-Urios et al., 2013). Both groups presented an increase in positive urgency and impairments in Stroop inhibition vs. healthy controls. The peak amount of cocaine use was inversely correlated with working memory and response inhibition performance (Albein-Urios et al., 2013). The authors argued that cocaine-specific elevations of negative urgency and deficits in working memory function may reflect “cocaine neurotoxicity” (Albein-Urios et al., 2013). This putative link to neurotoxicity is consistent with studies in both humans and animal models that showed cocaine-induced deficits in working memory and PFC dysfunction (George et al., 2007; Tomasi et al., 2007), the latter of which may impair the control over impulsive or compulsive substance use.

Cocaine-dependent patients with personality disorders had greater negative urgency, an increase in borderline beliefs, a decrease in inhibition and attention regulation, and a decrease in temporal pole gray matter volume compared with cocaine-dependent patients without personality disorders (Albein-Urios et al., 2013). The authors argued that patients with comorbid personality disorders present an increase in negative urgency and impairments in executive control, in addition to executive function deficits and impulsive traits that are seen in cocaine dependence (Albein-Urios et al., 2013). In a study that employed trait impulsivity measures and decision-making tasks in cocaine-dependent subjects and healthy controls, negative urgency distinguished individuals with cocaine dependence from healthy controls (Torres et al., 2013).

### Pathological Gambling

Data also support a role for negative urgency in pathological gambling. In a case-control study of 30 pathological gamblers and their respective controls, negative and positive urgency showed the greatest effect sizes of all impulsivity dimensions that discriminated groups (Michalczuk et al., 2011). Many other studies have observed increases in negative urgency in subjects with pathological gambling (Torres et al., 2013; Yan et al., 2016; Mick et al., 2017; Shakeel et al., 2019). A meta-analysis review that involved a total of 2,134 gamblers and 5,321 controls found that negative urgency had the greatest effect size of all impulsivity constructs (Maclaren et al., 2011). In a study of 1,002 Canadian adults, negative urgency, unique among impulsivity constructs, prospectively predicted subsequent problem gambling in both men and women (Farstad et al., 2015). Of the impulsivity constructs, negative urgency also uniquely predicted at-risk gambling (Yan et al., 2016), suggesting that it may be an early risk factor in the progression to addictive behavior. Negative urgency especially increased in a subgroup of pathological gamblers who presented high impairments in executive function and greater overall psychopathology (Mallorquí-Bagué et al., 2018b).

Particularly striking in Torres et al. (2013), negative urgency covaried uniquely with gambling overpathologization, supporting the hypothesis that negative urgency is a sign of overpathologization in addictive processes. Consistent with this hypothesis and supporting a trans-diagnostic role for negative urgency, studies have found that negative urgency is associated with not only the severity of gambling disorder (Savvidou et al., 2017; Steward et al., 2017) but also with cocaine dependence, tobacco addiction, and alcohol addiction (Albein-Urios et al., 2013; Spillane et al., 2013; VanderVeen et al., 2016; Daurio et al., 2018). Thus, negative urgency may be involved in the overpathologization of addiction in general, suggesting a common neurocircuitry basis. Also consistent with this trans-diagnostic perspective, negative urgency was higher in gamblers who smoked regularly vs. those who did not (Boothby et al., 2017). Negative urgency also longitudinally predicted worse treatment response in gamblers who received outpatient cognitive-behavior therapy, predicting low treatment compliance and greater relapse during treatment (Mallorquí-Bagué et al., 2018a; Mestre-Bach et al., 2019).

### Food

Negative urgency, in addition to other facets of impulsivity, is also high in disorders that involve the compulsive intake of palatable food, including food addiction as defined by the Yale Food Addiction Scale, and disorders with binge eating, including bulimia nervosa (Murphy et al., 2014; Ceccarini et al., 2015; Meule et al., 2015, 2017a,b; Pivarunas and Conner, 2015; de Vries and Meule, 2016; VanderBroek-Stice et al., 2017; Rose et al., 2018). Negative urgency is related more strongly to binge eating in patients with bulimia nervosa and adolescents with uncontrolled eating than to other aspects of impulsivity, such as sensation seeking, lack of planning, or lack of persistence (Fischer et al., 2008; Pearson et al., 2014; Booth et al., 2018). Furthermore, negative urgency was shown to predict binge eating symptoms prospectively from elementary school to middle school and in college students (Pearson et al., 2015) and adults (Farstad et al., 2015). Negative urgency was also associated with more frequent snacking in adolescents (Smith and Cyders, 2016; Coumans et al., 2018). A path analysis study of 315 patients with eating disorders on the binge spectrum found that low self-directedness and emotional regulation were broadly associated with eating psychopathology, whereas negative urgency was uniquely associated with food addiction (Wolz et al., 2017). From a treatment standpoint, a multicomponent behavior therapy intervention in obese adolescents reduced body mass index relative to the degree to which it reduced negative urgency (Delgado-Rico et al., 2012). From a comorbidity perspective, negative urgency also predicted alcohol use problems in women with disordered eating (Fischer et al., 2007).

### Summary and Future Directions

In summary, clinical and community-based findings support the hypothesis that negative urgency plays a transdiagnostic role in addiction. It is increased across alcohol, tobacco, cocaine, gambling, and food addictive disorders, especially with regard to problematic and hazardous use. In many studies, negative urgency was associated with measures of addiction severity and comorbid addiction or other psychopathology. Several findings indicate a greater or unique role for negative urgency compared with other recognized constructs of impulsivity, including positive urgency. Negative urgency was associated with negative emotions, impairments in executive control, and substance use in many studies, but more work is needed to clarify its mediating vs. moderating role in the relationships among these constructs and the degree to which it operates as an antecedent risk factor vs. emerging consequence of addiction. Initial work suggests that negative urgency may be an early risk factor, but it may also be a useful target for treatment or predictor or indicator of treatment response, areas that warrant further study. The etiology of antecedent individual differences in negative urgency also remains to be determined. Studies have shown that negative urgency is a relevant construct in both men and women (Smith and Cyders, 2016). No consistent gender differences have been reported at the level of negative urgency (Smith and Cyders, 2016), but negative urgency may still play differential roles between genders for specific behaviors (Davis-Becker et al., 2014). Moreover, levels of negative urgency differ according to pubertal status (i.e., increasing with puberty; Gunn and Smith, 2010; Pearson et al., 2010). Thus, the role of negative urgency in known gender and developmental differences in addiction vulnerability remain to be more fully explored. Finally, new work that uses ecological momentary assessment or similar approaches is needed to test the model that is proposed herein that impulsive and eventually compulsive substance seeking and use more often follow abstinence- or environment-associated distress in people who are high in negative urgency.

## Neurocircuitry Implicated in Negative Urgency and Addiction

**Figure 2:**
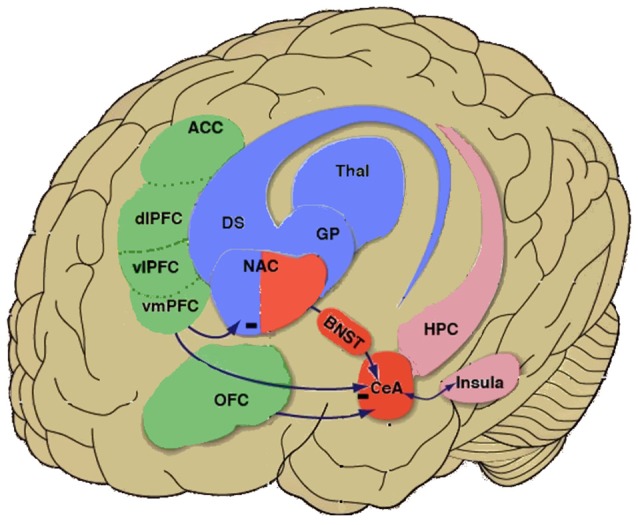
Negative urgency circuitry in the neurobiology of addiction. Simplified inter-relationships are shown between higher-order cortical regions [green shading: orbitofrontal cortex (OFC) and compartments of the prefrontal cortex (PFC), including the anterior cingulate cortex (ACC), dorsolateral PFC (dPFC), ventrolateral PFC (vPFC) and ventromedial PFC (vmPFC)] that regulate activity of the extended amygdala (red shading: central nucleus of the amygdala, bed nucleus of the stria terminalis, portion of nucleus accumbens shell) and basal ganglia (blue shading: including nucleus accumbens core, dorsal striatum, globus pallidus). Negative urgency is hypothesized to reflect a vulnerability to extreme negative affect that impairs the efficacy of higher-order inhibitory control from such regions as the OFC and ventromedial PFC over drug-taking and drug-seeking behaviors that are subserved by the extended amygdala and basal ganglia. Negative urgency is also hypothesized to reflect alterations of cortico-amygdalar and cortico-striatal modulation by the insular cortex (representing interoceptive state and context) and other prefrontal cortical regions, including the prelimbic cortex and ACC. Note that regions are illustrated heuristically and are not intended to be neuroanatomically precise. Reprinted from Koob et al. (2013), with permission from Elsevier.

Neurobiological data on negative urgency are limited to date, but negative urgency has been hypothesized to reflect impairments in the “top-down” cortical control over both basal ganglia and extended amygdala function (Figure 2). This topic was very recently reviewed in detail (Um et al., 2019b), so we only briefly discuss key findings here. Most, if not all, of the data are derived from human imaging studies. Deficient top-down control has been hypothesized to reflect a loss of control over pathological habits that involve basal ganglia and extended amygdala processing (Robbins and Everitt, 1999; Everitt and Robbins, 2005; George et al., 2007; Belin et al., 2013) and greater attention to, incentive salience of, or cognitive resource interference from emotion-evoking stimuli. Consequently, in the presence of negative emotion, there is a reduction of inhibitory control over potentially detrimental actions and habits, the latter reflecting increased behavioral control by the dorsolateral striatum (Everitt and Robbins, 2005; Belin and Everitt, 2008; Belin et al., 2013; Giuliano et al., 2019). These biases putatively reflect alterations of the structure, function, or connectivity of orbitofrontal cortex (OFC)/ventromedial prefrontal cortex (vmPFC) projections to the basal ganglia and extended amygdala (Cyders and Smith, 2008; Robbins et al., 2012; Smith and Cyders, 2016; Figure 2).

Supporting this hypothesis, trait urgency was related to the amplitude of resting-state low-frequency fluctuations in the lateral OFC and vmPFC in healthy volunteers (Zhao et al., 2017). Trait negative urgency also predicted an increase in activation of the vmPFC in response to an alcohol odor cue in social drinkers and mediated the association between activation of the vmPFC and alcohol craving and problematic drinking (Cyders et al., 2014a). Similarly, negative urgency predicted greater OFC and amygdala activation in response to negative visual stimuli and mediated the relationship between activation and risky behavior (Cyders et al., 2015). Additionally, negative urgency was also associated with resting-state and inhibitory task-related activation (Go/No-Go or gambling tasks) in other structures that subserve self-regulation and decision making under risk. These included the dorsolateral and ventrolateral PFC, anterior insula, and anterior and posterior cingulate (Clark et al., 2008; Xue et al., 2010; Hoptman et al., 2014; Chester et al., 2016; Zhao et al., 2017). Greater insula activation prospectively predicted real-world substance use in subjects who were high in negative urgency (Chester et al., 2016). Negative urgency also predicted greater increases in medial PFC (mPFC) activity during anticipation in a delayed incentive task (Weiland et al., 2014). Finally, in patients with schizophrenia, urgency was associated with a reduction of cortical thickness in such structures as the vmPFC, orbitofrontal and inferior frontal gyri, and rostral anterior cingulate cortex (Hoptman et al., 2014). As discussed above, cocaine-dependent subjects with comorbid personality disorders had more negative urgency, more intense borderline beliefs, inferior response inhibition and attention regulation, and less gray matter in the right temporal pole compared with cocaine-dependent individuals without these comorbidities (Albein-Urios et al., 2013). Others have shown a wide distribution of lower gray matter volume in the PFC, insula, and amygdala-temporal lobe in cocaine-dependent subjects compared with healthy controls, with a correlation between greater trait impulsivity (i.e., a lack of premeditation and negative urgency) and lower gray matter volume in the left inferior/middle frontal gyrus in cocaine-dependent individuals (Moreno-López et al., 2012).

Neurochemically, negative urgency may reflect deficient 5-hydroxytryptamine (5-HT) and lower dopamine activity in the OFC and vmPFC (Floresco and Tse, 2007; Cyders and Smith, 2008), leading to less inhibition of both basal ganglia- and extended amygdala-subserved impulses. Accordingly, a composite polygenic 5-HT score predicted alcohol problems *via* an increase in negative urgency and not *via* other measures of impulsivity (Carver et al., 2011; Wang and Chassin, 2018). Evidence of lower 5-HT activity and responsiveness is likewise seen in bulimia nervosa with regard to the presence and severity of binge symptoms (Cyders and Smith, 2008). Genetic variation of the *GABRA2* subunit, which encodes the γ-aminobutyric acid (GABA) receptor α2 subunit and is related to alcohol use problems *via* urgency, has also been linked to alterations of insula activation responses (Villafuerte et al., 2012) and dorsolateral PFC GABA concentrations (Boy et al., 2011).

Traditionally, negative urgency has been conceptualized as a stable dispositional antecedent that potentiates responses to extreme situational distress (e.g., Engel et al., 2007; Fischer et al., 2018). Consistent with this view, negative urgency predicted greater subsequent mood changes, alcohol cue-induced craving, and intravenous alcohol self-administration after negative mood induction (VanderVeen et al., 2016) and an increase in negative affect and relative reinforcing value after laboratory stressors (Owens et al., 2018). From the “dark side” perspective, negative urgency is viewed as a trait that increases the likelihood of relapsing during opponent-process- or life stress-associated distress (Ahmed and Koob, 2005).

Negative urgency, however, is not strictly trait-like. Situational factors can impact inhibitory signals from the OFC/vmPFC to the amygdala (Silbersweig et al., 2007). Relevant to drug addiction, both cues (positive and negative), the onset of negative emotional states, and any combination thereof can trigger the reinstatement of drug seeking. Accordingly, an increase in substance cue reactivity is seen in the hypothesized negative urgency network (i.e., basal ganglia, amygdala, OFC, cingulate cortex, vmPFC, dorsolateral PFC, and anterior insula) in people with substance use disorders and predicts relapse (Heinz et al., 2009; Goudriaan et al., 2010; Engelmann et al., 2012; Mainz et al., 2012; Jasinska et al., 2014).

Moreover, environmental history can elicit enduring changes in urgency. For example, childhood abuse persistently increased amygdala activation and reduced prefrontal cortical control over amygdalar responses (Teicher et al., 2003) and is well documented to predispose individuals to drug addiction (Choi et al., 2017). Furthermore, effective psychological interventions for obesity and gambling disorder have been reported to reduce negative urgency (Delgado-Rico et al., 2012; Garcia-Caballero et al., 2018).

## Negative Urgency and the Dark Side of Addiction

A novel hypothesis that is proposed herein is that opponent-process adaptations to alcohol, tobacco, and other substances of abuse may also increase both negative affect and negative urgency. Decreases in striatal dopamine D_2_ receptor availability in alcohol-addicted subjects were correlated with lower glucose metabolism in frontal cortical regions that underlie inhibitory control, such as the dorsolateral and anterior cingulate cortices (Volkow et al., 2007). Importantly, these relationships are not seen in non-AUD controls, consistent with the hypothesis that they reflect circuitry changes. Perhaps accordingly, negative urgency also predicted greater caudate responses to alcohol-related images in alcohol-dependent individuals (Chester et al., 2016). Similarly, in pathological gamblers, higher negative urgency was correlated with lower striatal D_2_ receptor availability, indexed by [^11^C]-raclopride binding potential (Clark et al., 2012).

As noted above, the increase in substance cue reactivity that is seen in prefrontal-basal ganglia and prefrontal-amygdala circuits in subjects with substance use disorders predicts relapse and may reflect adaptations within negative urgency circuits. These circuits may involve not only reward processing, as is often interpreted (e.g., Stice et al., 2015; Schulte et al., 2016; Winter et al., 2017), but also stress processing (Koob and Schulkin, 2018). This perspective is reinforced by the finding that central nucleus of the amygdala activation maintains habitual, drug-seeking behavior *via* dorsolateral striatum activation (Murray et al., 2015). From a “dark side” perspective, negative urgency forms another pathway for impulsivity deficits to continue throughout the addiction cycle, not being simply limited to positive urgency, reward, and basal ganglia function.

## Animal Model of Negative Urgency?

In an intriguing attempt to bridge human self-reports and behavioral measures of negative urgency to animal models, some groups have attempted to back-translate negative urgency concepts to rats. One group showed that human subjects who scored high in negative urgency presented more behavioral responding and greater frustration following unexpected reward omission in a monetary-based task vs. subjects who scored lower in negative urgency (Gipson et al., 2012). Similarly, they found that rats exhibited an increase in operant responses for intravenous amphetamine or sucrose pellets after unexpected reward omission. The results suggested that impulsive behavior that is engendered by the unexpected omission of reward may represent a valid behavioral model of negative urgency that can be linked to substance abuse (Gipson et al., 2012). In a follow-up study that used the same reward omission task to determine the neurochemical bases of the reward omission effect in this model of negative urgency, contingent responding was higher following the omission of an expected reward than following the delivery of an expected reward (Yates et al., 2015). Dopamine and 5-HT uptake were measured in individual rats using synaptosomes that were prepared from the nucleus accumbens, dorsal striatum, mPFC, and OFC. The V_max_ values for the dopamine transporter in the nucleus accumbens and serotonin transporter in the OFC were positively correlated with negative urgency scores, suggesting that mood-based impulsivity (i.e., negative urgency) is associated with greater dopamine transporter function in the nucleus accumbens and serotonin transporter function in the OFC (Yates et al., 2015). Similarly, Cifani et al. (2009) performed a series of studies and found that the frustrative nonreward of being placed in a context where a previously available preferred food could be seen and smelled but no longer eaten elicited behavioral and neuroendocrine signs of stress and subsequently greater palatable food self-administration and binge eating (Cifani et al., 2009; Piccoli et al., 2012) *via* mechanisms that involved extended amygdala CRF activation (Micioni Di Bonaventura et al., 2014; Pucci et al., 2016). These results highlight the power of the ecologically valid challenges of reward omission and frustrative nonreward to drive an increase in use. The models also are consistent with the increasing trend for preclinical models to recapitulate diagnostic and translationally-meaningful symptoms of substance use disorders (Belin-Rauscent et al., 2016). To develop the models further within a negative urgency framework, remaining to be shown is that the use is “driven forward” in an impulsive manner, meaning: (i) rapidly/suddenly; (ii) with prioritization of immediate vs. later outcomes (e.g., in a delayed-discounting task framework; Herman et al., 2018); and (iii) in a risky fashion, without apparent behavioral consideration given to possible negative outcomes. Ultimately, the latter aspect may progress to compulsive behavior, in which use commences not only hastily and without forethought of possible negative outcomes but even despite recognized and experienced negative outcomes (e.g., if drug-reinforced responses are instead punished or presented in other similar approach-avoidance conflict frameworks; Pelloux et al., 2007; Jonkman et al., 2012).

Economidou et al. (2009) have previously shown that antecedent high impulsivity, defined by a 5-choice serial reaction time task, predicted cocaine relapse after punished responding. Similarly, Belin et al. (2008), Ansquer et al. (2014), and Murray et al. (2014) showed that high impulsivity in the 5-choice serial reaction time task prospectively predicted the development of compulsive cocaine self-administration and compulsive adjunctive behavior, in a manner mitigated by the norepinephrine transporter inhibitor atomoxetine. Here, we propose that a negative mood-driven measure of urgent impulsivity (e.g., greater reward omission-induced shifts toward immediate vs. later rewards or the greater reward omission-enhancement of substance seeking/use even in an exposed or otherwise risky context) may similarly predict the development of compulsive drug use or relapse.

## Conclusions

The transition from drug use to addiction is accompanied by the downregulation of brain reward circuitry and the enhancement of “antireward”/stress circuitry that involves an opponent-process “dark side” of emotional dysregulation. Negative urgency may reflect impairments in “top-down” cortical-to-basal ganglia and cortical-to-extended amygdala processing, leading to a reduction of inhibitory control over potentially detrimental actions. Such impairments may yield heightened “bottom-up” basal ganglia and extended amygdala processing, leading to greater attention to incentive salience, pathological habits, or cognitive resource interference from emotion-evoking stimuli. Limited data to date, almost exclusively in humans, reflect alterations of the structure, function, or connectivity of orbitofrontal/ventromedial prefrontal cortical–basal ganglia and extended amygdala projections and support the hypothesis that the resulting negative urgency facilitates the transition to compulsive drug seeking and may even help maintain compulsive drug seeking. The further development of animal models and human experiments to better study the most critical aspects of negative urgency and identify molecular loading on specific neurochemical circuits that convey vulnerability and resilience to compulsive drug seeking *via* negative urgency are charges for the future.

## Glossary

### a-process

The a-process represents the initial positive hedonic or mood impact of a stimulus. In summation with the subsequent, opponent, counter-regulatory b-process, it yields a net affective stimulus (state). An individual who still experiences a positive hedonic mood state from a drug of abuse is hypothesized to retain a predominant a-process and experience positive reinforcement when using a substance of abuse (Koob and Le Moal, 2001).

### Allostasis

The process of achieving stability through change (Koob and Le Moal, 2001).

### Allostatic Load

The cost to the brain and body of the deviation, accumulating over time, and reflecting in many cases pathological states and accumulation of damage (Koob and Le Moal, 2001).

### Allostatic State

A state of chronic deviation of the regulatory system from its normal (homeostatic) operating level (Koob and Le Moal, 2001).

### Antireward

A concept based on the hypothesis that there are brain systems whose function is to limit reward when triggered by excessive activity in the reward system. Both within-system and between-system neuroadaptations may underlie antireward adaptations in addiction.

### Between-System Neuroadaptation

A between-system neuroadaptation is defined as a circuitry change, in which one circuit (i.e., stress or anti-reward circuits) becomes activated by another circuit (i.e., the reward circuit; Koob, 2004).

### b-process

The b-process represents the counter-regulatory, opponent-process response to the initial activating a-process. In summation with the prior, initial a-process, it yields a net affective stimulus (state). A homeostatic b-process that simply balances the activational process (a-process) would restore the initial emotional equilibrium set point. In the affective dysregulation, allostasis model, however, the b-process does not balance the activational process but rather shows residual hysteresis with repeated engagement. This creates a progressively greater allostatic state and a persistent, net negative affective stimulus when drug use stops, experienced as withdrawal and, later, protracted abstinence. An individual in this allostatic state is hypothesized to experience negative reinforcement, with partial relief from the negative state when using a substance of abuse (Koob and Le Moal, 2001).

### Compulsivity

A behavioral predisposition to experience and act upon irresistible urges. Compulsive behaviors are often experienced as outside of one’s control, intrusive, and unwanted. They can be operationally defined as perseverative responding in the face of adverse consequences or in the face of incorrect outcomes (Koob, 2014).

### Impulsivity

A behavioral predisposition toward rapid, unplanned reactions to stimuli without regard to the possible negative consequences of these reactions to the self or others. Operationally, impulsivity is often measured as a bias toward immediate smaller vs. delayed larger rewards and the inability to inhibit or alter a course of action (Koob, 2014).

### Incentive Salience

Through the process of conditioning, previously neutral stimuli become linked to natural or drug reinforcers and acquire the ability to engender or increase the motivation to seek the reinforcer (Koob and Le Moal, 2006).

### Negative Urgency

The behavioral predisposition to act rashly and impulsively when in extreme distress (Cyders and Smith, 2008).

### Positive Urgency

The behavioral predisposition to act rashly and impulsively when in an extremely positive mood state, such as euphoria (Cyders and Smith, 2008).

### Within-System Neuroadaptation

Within-system neuroadaptations are the process by which the primary cellular response element to the drug adapts to neutralize the drug’s effects. Thus, within-system neuroadaptations occur within the neurocircuits and neurochemical systems that are initially engaged by the rewarding substance to elicit its rewarding effects, or a-process (Koob, 2004). Persistence of these opposing effects after the drug disappears produces adaptation.

## Author Contributions

EZ and GK contributed to writing the manuscript equally.

## Conflict of Interest Statement

EZ and GK are inventors on a patent filed for CRF_1_ receptor antagonists (USPTO application no. 2010/0249138).
