# Corrigendum: Impulsivity Derived From the Dark Side: Neurocircuits That Contribute to Negative Urgency

**DOI:** 10.3389/fnbeh.2019.00188

**Published:** 2019-08-27

**Authors:** Eric P. Zorrilla, George F. Koob

**Affiliations:** ^1^Department of Neuroscience, The Scripps Research Institute, La Jolla, CA, United States; ^2^Neurobiology of Addiction Section, Intramural Research Program, National Institute on Drug Abuse, Baltimore, MD, United States

**Keywords:** negative urgency, impulsivity, compulsive drug use, negative affect, withdrawal, substance or alcohol use disorder, orbitofrontal cortex, extended amygdale

In the original article there was an error. The reference for Um et al. (2019) was incorrectly cited.

A correction has been made to the section ***Negative Urgency and Addiction: Tobacco***, ***Alcohol, Cocaine, Pathological Gambling***, ***and Food*** subsection ***Alcohol***, paragraph seven:

“A study of 675 community-dwelling adults in the Rockland Project used structural equation modeling path analysis to evaluate the mediating vs. moderating roles of urgency in the relationship between depression and problematic alcohol or cannabis use. Negative urgency, not positive urgency, was a unique mediator of the relationships between depressive symptoms and both problematic alcohol use and problematic cannabis use. Additionally, negative urgency moderated the relationship between depressive symptoms and problematic cannabis use. Specifically, at low levels of negative urgency, depressive symptoms predicted less problematic cannabis use, whereas at high levels of negative urgency, depressive symptoms predicted greater cannabis use. The authors concluded that despite being statistically correlated with each another, negative and positive urgency had distinct influences on the relationship between depressive symptoms and alcohol and cannabis use, with negative urgency having unique predictive significance (Um et al., 2019a).”

Additionally, a correction has been made to the section ***Neurocircuitry Implicated in*** ***Negative Urgency and Addiction***, paragraph one:

“Neurobiological data on negative urgency are limited to date, but negative urgency has been hypothesized to reflect impairments in the “top-down” cortical control over both basal ganglia and extended amygdala function (Figure 2). This topic was very recently reviewed in detail (Um et al., 2019b) so we only briefly discuss key findings here. Most, if not all, of the data are derived from human imaging studies. Deficient top-down control has been hypothesized to reflect a loss of control over pathological habits that involve basal ganglia and extended amygdala processing (Robbins and Everitt, 1999; Everitt and Robbins, 2005; George et al., 2007; Belin et al., 2013) and greater attention to, incentive salience of, or cognitive resource interference from emotion-evoking stimuli. Consequently, in the presence of negative emotion, there is a reduction of inhibitory control over potentially detrimental actions and habits, the latter reflecting increased behavioral control by the dorsolateral striatum (Everitt and Robbins, 2005; Belin and Everitt, 2008; Belin et al., 2013; Giuliano et al., 2019). These biases putatively reflect alterations of the structure, function, or connectivity of orbitofrontal cortex (OFC)/ventromedial prefrontal cortex (vmPFC) projections to the basal ganglia and extended amygdala (Cyders and Smith, 2008; Robbins et al., 2012; Smith and Cyders, 2016; Figure 2).”

The authors apologize for this error and state that this does not change the scientific conclusions of the article in any way. The original article has been updated.

## References

[B1] Belin D. Belin-Rauscent A. Murray J. E. Everitt B. J. (2013). Addiction: failure of control over maladaptive incentive habits. Curr. Opin. Neurobiol. 23, 564–572. 10.1016/j.conb.2013.01.025 23452942

[B2] Belin D. Everitt B. J. (2008). Cocaine seeking habits depend upon dopamine-dependent serial connectivity linking the ventral with the dorsal striatum. Neuron 57, 432–441. 10.1016/j.neuron.2007.12.019 18255035

[B3] Cyders M. A. Smith G. T. (2008). Emotion-based dispositions to rash action: positive and negative urgency. Psychol. Bull. 134, 807–828. 10.1037/a0013341 18954158 PMC2705930

[B4] Everitt B. J. Robbins T. W. (2005). Neural systems of reinforcement for drug addiction: from actions to habits to compulsion. Nat. Neurosci. 8, 1481–1489. 10.1038/nn1579 16251991

[B5] George O. Ghozland S. Azar M. R. Cottone P. Zorrilla E. P. Parsons L. H.. (2007). CRF-CRF_1_ system activation mediates withdrawal-induced increases in nicotine self-administration in nicotine-dependent rats. Proc. Natl. Acad. Sci. U S A 104, 17198–17203. 10.1073/pnas.0707585104 17921249 PMC2040467

[B6] Giuliano C. Belin D. Everitt B. J. (2019). Compulsive alcohol seeking results from a failure to disengage dorsolateral striatal control over behavior. J. Neurosci. 39, 1744–1754. 10.1523/jneurosci.2615-18.2018 30617206 PMC6391574

[B7] Robbins T. W. Everitt B. J. (1999). Drug addiction: bad habits add up. Nature 398, 567–570. 10.1038/19208 10217139

[B8] Robbins T. W. Gillan C. M. Smith D. G. de Wit S. Ersche K. D. (2012). Neurocognitive endophenotypes of impulsivity and compulsivity: towards dimensional psychiatry. Trends Cogn. Sci. 16, 81–91. 10.1016/j.tics.2011.11.009 22155014

[B9] Smith G. T. Cyders M. A. (2016). Integrating affect and impulsivity: the role of positive and negative urgency in substance use risk. Drug Alcohol Depend. 163, S3–S12. 10.1016/j.drugalcdep.2015.08.038 27306729 PMC4911536

[B10] Um M. Hershberger A. R. Cyders M. A. (2019a). The relationship among depressive symptoms, urgency, and problematic alcohol and cannabis use in community adults. Addict. Behav. 88, 36–42. 10.1016/j.addbeh.2018.08.009 30130721 PMC6217802

[B11] Um M. Whitt Z. T. Revilla R. Hunton T. Cyders M. A. (2019b). Shared neural correlates underlying addictive disorders and negative urgency. Brain. Sci. 9:E36. 10.3390/brainsci9020036 30744033 PMC6406305

